# Light People: Professor Thomas G. Brown

**DOI:** 10.1038/s41377-022-00997-0

**Published:** 2022-10-18

**Authors:** Hui Wang

**Affiliations:** Changchun Institute of Optics, Fine Mechanics and Physics, Chinese Academy of Sciences, 3888 Dong Nan Hu Road, Changchun, 130033 China

**Keywords:** Polymers, Imaging and sensing

## Abstract

What’s the first thing that comes to your mind when it comes to the University of Rochester? It is the first optical education program in the USA, and one of the three most respected optical education and research centers programs in the country. Nobel Prize Laureates such as Gerard Mourou and Donna Strickland have completed major works here. And today we’re going to introduce Prof. Thomas G. Brown, Director of the University of Rochester’s Institute of Optics.

Prof. Brown has many scientific achievements to his name, including introducing the term “cylindrical vector beam” to describe unique and unconventional polarization states. He has ample experience in both scientific research and industry development, and now is an inspiring mentor to young students and researchers, and a respected chief editor of academic journals. Please follow the Light People to the Institute of Optics at the University of Rochester, learn about Thomas G. Brown’s vision for the future, and explore his colorful life path, which will bring you new inspirations.

## Abstract (web summary)

Prof. Thomas G. Brown from the University of Rochester introduced the term “cylindrical vector beam” to describe unique and unconventional polarization states.

## Graphical abstract

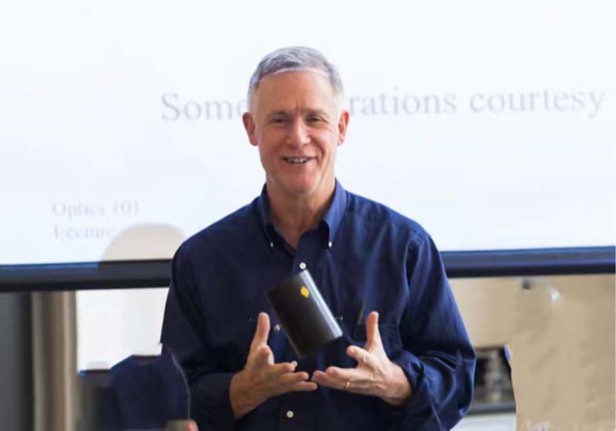

**Biography** Thomas G. Brown has been on the faculty of the Institute of Optics since July of 1987, has held the rank of full professor since 2008 and is currently the director of the Institute of Optics and a Mercer Brugler Distinguished Teaching Professor. Professor Brown is a Fellow of Optica and SPIE, is Editor in Chief of the *Journal of Modern Optics*, and serves as chair of the annual multidimensional microscopy conference (Photonics West). He was the founding director of the Robert E. Hopkins Center for Optical Design and Engineering, the architect of the optical engineering curriculum at the Institute of Optics, served as a program co-chair for the centennial program of Optica and is former president and honorary member of the Rochester Local Chapter of Optica.

**Figure:**
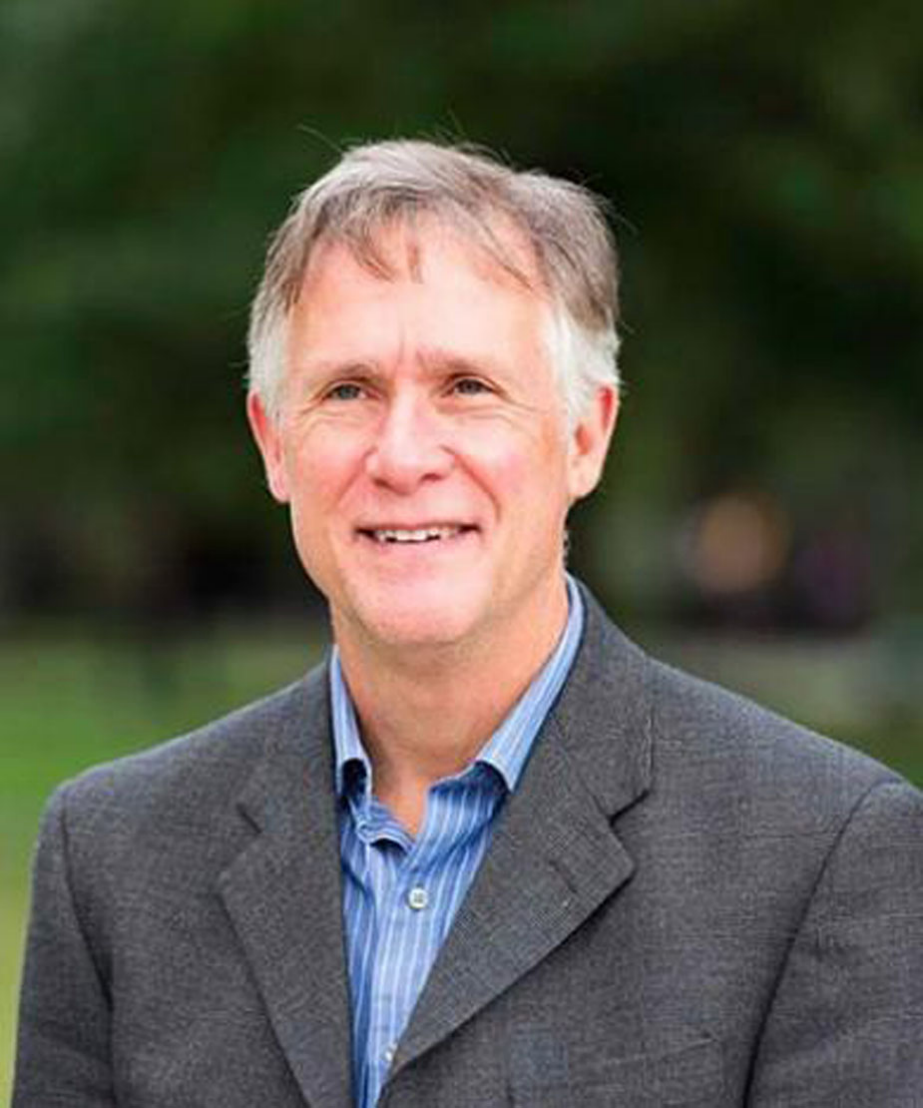

Professor Brown began his work in optics and optoelectronics in 1978 as an optical fiber systems designer at GTE Laboratories. He has had consultancies and technical collaboration with companies such as Qualcomm, IBM, Corning Inc., ABB Kent-Taylor, Amp, Rockwell, Rochester Gas and Electric, and Emerson Corporation, along with several law firms and many of the Industrial Associates of the Institute of Optics.

Professor Brown is frequently asked to provide expert consulting in a wide range of areas in optical systems, photonics, and the application of light-based technologies to a wide range of manufacturing applications, including emerging areas of photonics.

**1. Could you briefly introduce your current research and your latest progress?**

Prof. Brown: I have three areas of current research: (1)stress-engineered optics (SEOs) and how they can be used in microscopy, polarimetry and in the study of orbital angular momentum (OAM) beams; (2) metrology of photonic integrated circuits with special emphasis on measuring the in situ polarization state within a silicon or silicon nitride waveguide; (3) the use of reflection holograms in freeform optical metrology.

**Figure:**
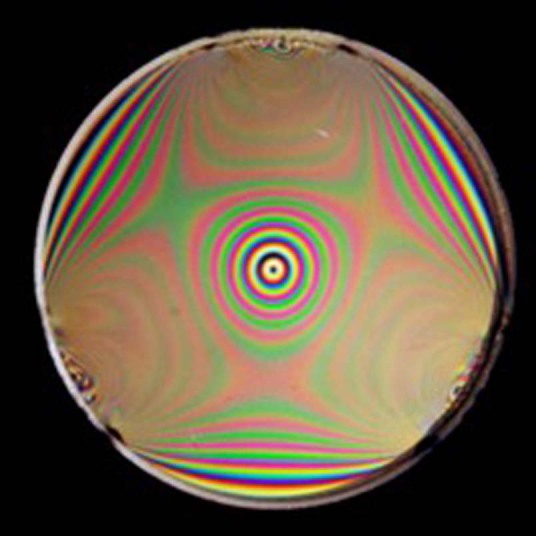
Image of a stressed optic viewed through circular polarizers. The bright rings correspond to optical vortices. Ashan Ariyawansa

**2. You and your colleagues introduced the idea of a full Poincare beam, a fully correlated beam that contains every possible polarization state, you coined the term ‘Cylindrical Vector Beam’ in analyzing the tight focusing properties of Radial and Azimuthally polarized beams. This is a major achievement, so could you brief us on this project please? What is the current research on this technology and how is it being used?**

Prof. Brown: In the year 2000, we chose the term ‘Cylindrical Vector Beams’ to distinguish beams having radial (spoke-like) and azimuthal (tangential) polarization about the beam axis with perfect cylindrical symmetry. We began to study the focusing properties of these beams in an effort parallel to that of Gerd Leuchs and his group at the Max Planck Institute for Light. Both groups quickly identified remarkable properties of these beams under very tight focusing conditions. With Lukas Novotny (then on the faculty at Rochester) we were able to demonstrate that these optical fields could identify molecular orientation in single molecule fluorescence measurements. We introduced stress-engineered optics (SEO) as one way of producing beams of varying polarization in situations where liquid crystal optics is unavailable or impractical. Along with Prof. Miguel Alonso, we discovered that the Full Poincare beam had intriguing physical and mathematical properties. The use of SEOs also revealed new ways of performing single shot Stokes polarimetry.

**Figure:**
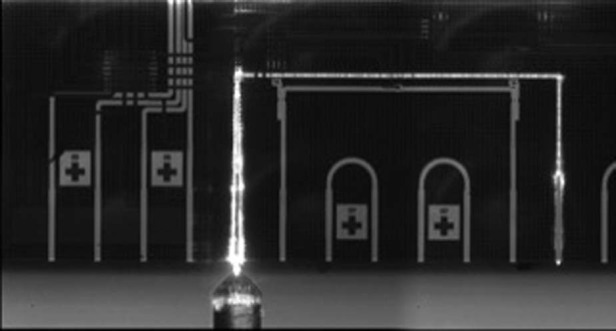
Short wave infrared (SWIR) image of a photonic integrated circuit fabricated at AIM Photonics as part of Prof. Brown’s research

**3. The Institute of Optics of the University of Rochester is not only the first US educational program devoted solely to the research and teaching of optics, but is also one of the top three optics research centers in the USA currently. As the head of this venerable institute, can you give us a history lesson please and tell us why Rochester was chosen to be the home to the first US optics institute? What in your opinion are your institute’s unique appeals and features compared with the College of Optical Science of the University of Arizona and the College of Optics and Photonics of the University of Central Florida? And also could you let us in on some of the future development plans of your institute?**

Prof. Brown: The Institute of Optics is the USA’s original academic program and research institute dedicated to the study of optics. It was established in 1929 to meet a national need to train optical scientists and engineers on US soil. We are very proud of the fact that graduates of the Institute were instrumental in establishing the Optical Sciences Center in Tucson, that became the James C Wyant College of Optical Sciences. Our unique appeals include: A relatively small educational center connected to a very large network of local scientific and industrial activity in optics. As a result, students get to know one another and the professors very well, but also are able to connect to others within our larger network. This network includes optics researchers in both other academic departments such as physics, chemistry and electrical/computer engineering, R&D centers (e.g. the Laboratory for Laser Energetics and the Center for Visual Science) and the members of our Industrial Associates Program. Our development plans include expansion of our core faculty, expansion of our Industrial Associates Program, and ongoing innovation in our Hybrid Optics Masters’ Education (HOME) program.

**Figure:**
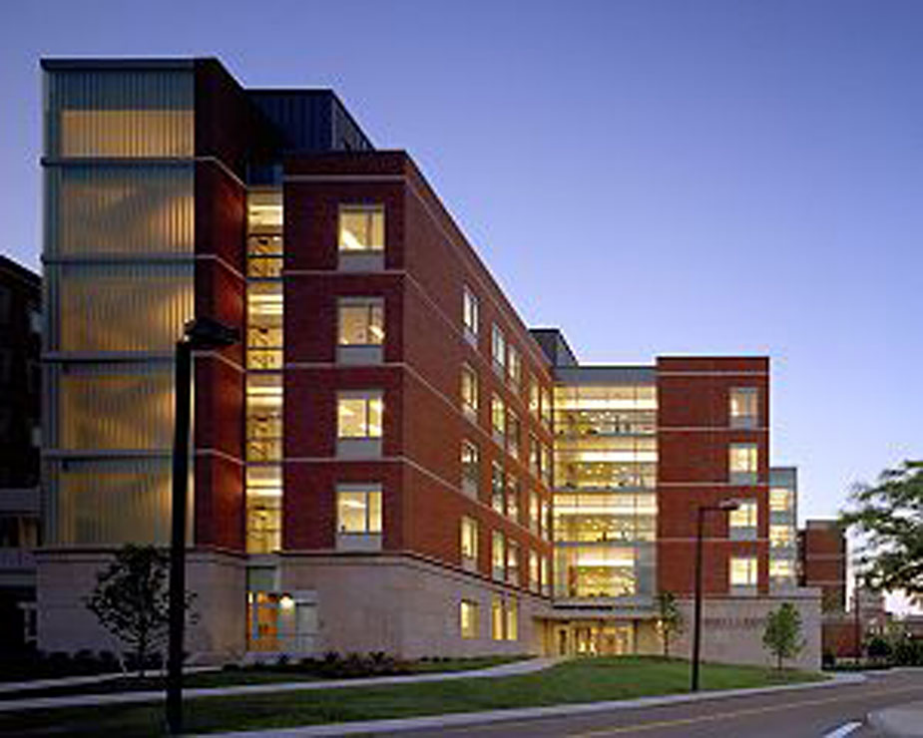
An evening view of The Institute of Optics at Goergen Hall (University of Rochester)

**4. You are the founding director of the Robert E. Hopkins Center for Optical Design and Engineering, so could you briefly introduce this center? What do you think of the center’s development today? What expectations do you have for it in the future?**

Prof. Brown: The Hopkins Center was established through a generous gift by John Bruning, an optical engineer and entrepreneur, to honor Robert Hopkins, a PhD graduate and former director of the Institute of Optics. Its mission is to bring modern design, fabrication and testing tools into undergraduate and masters level education. Our lens design and coating design courses taught by Profs. Bentley and Kruschwitz are among the best and most unique in the world. It is now under the leadership of Prof. Jannick Rolland, who has pioneered the field of freeform optics and has established the Center for Freeform Optics at the University of Rochester and the University of North Carolina at Charlotte.

**5. Nobel Prize laureates in Physics Gérard Mourou and Donna Strickland have both studied or worked at the University of Rochester, as well as many other respected academics in the field of optics research. What contributions do you think the University of Rochester made to their success?**

Prof. Brown: The Nobel prize winning work was accomplished while Donna was a PhD student at the Institute of Optics, working in the research group of Gérard Mourou at the Laboratory for Laser Energetics. The work was very much a combination of the energy, creativity and hard work of Strickland and Mourou along with an environment that combines a thorough understanding of classical optics and optical engineering with optical physics.

**6. You are the editor-in-chief of the** ***Journal of Modern Optics*** **(JMO), what do you think are the best and worst parts about being responsible for a journal?**

Prof. Brown: The best part is being exposed to the breadth of research being done all over the world. JMO is a very international journal—we have contributions from every country that has optics research. The challenging part is maintaining high writing standards for a journal that has so many contributors (authors and referees) for whom English is a second language.

**7. You have authored over 95 publications, 3 book chapters, edited the four-volume Optics Encyclopedia and one of your papers was ranked number 6 of the top ten most cited papers by** ***Optics Express*** **on its 25**^**th**^ **birthday. As a journal editor and a successful published writer, what advice or suggestions do you have for young researchers on academic paper writing?**

Prof. Brown: My advice is to look for original ideas that have not yet been tested and to avoid simply imitating work being published in so-called high impact journals. Our work on cylindrical vector beams was published in *Optics Express* before it even had a measurable impact factor. The work of Strickland and Mourou was published in journals that had a solid reputation but didn’t over-emphasize citation counts. A good paper will be read and appreciated regardless of where it is published.

**8. You served as chair of the annual multidimensional microscopy conference (Photonics West), would you pleases give young researchers some advice on giving academic presentations?**

Prof. Brown: Always take time at the beginning to educate your audience and explain why the topic is important and/or interesting to you. You should be able to say, of each slide, ‘This is important (or interesting) because …’. If it is not important or interesting perhaps you don’t need to include it.

**9. You are actively involved in many international academic organizations such as: Optica, SPIE, the Materials Research Society (MRS), and the American Physical Society (APS). What do you think you have gained from participating in these organizations?**

Prof. Brown: Most of my activity has been with Optica and SPIE—both are extraordinary organizations for both professional networking and student mentoring. They bring together optical scientist and engineers that work in different subfields and otherwise might never meet. Both APS and MRS have also been important, although earlier in my career.

**10. You have technical collaborations with companies such as Qualcomm, IBM, Corning Inc., ABB Kent-Taylor, Amp, along with several law firms and many of the Industrial Associates of the Institute of Optics. What do you think should be the relationship between scientific research and commercialization?**

Prof. Brown: I think it is helpful for researchers to know what it takes to solve problems in the marketplace. Solutions involving cutting edge science make very good publications, but it is often the exquisitely simple and robust solutions that contribute the most to high quality commercial and medical technology.

**Figure:**
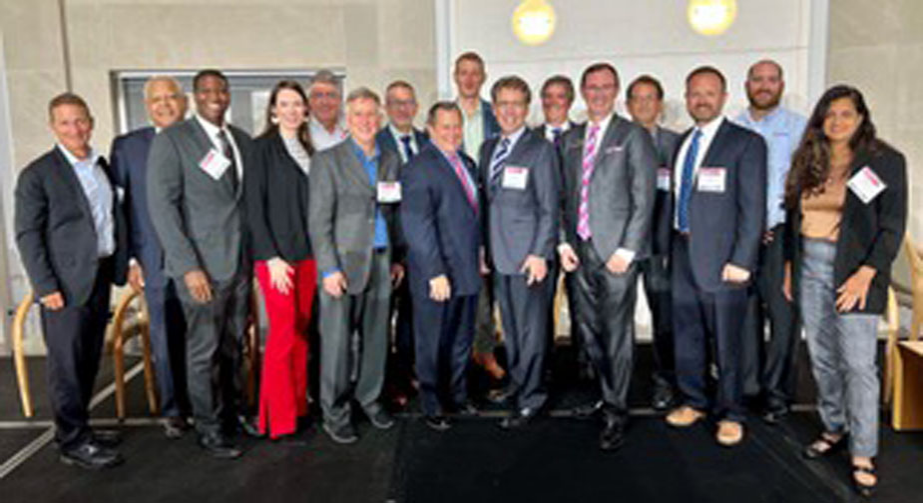
Prof. Brown in a Rochester delegation to Washington DC, participating in a Photonics Industry Summit

**11. As a tutor, you have trained many students. What abilities do you most want in your students?**

Prof. Brown: The most important qualities are hard work and a desire to learn beyond just what you need for the next step in your career. Things you learned 20 years ago might be very important in your job today.

**Figure:**
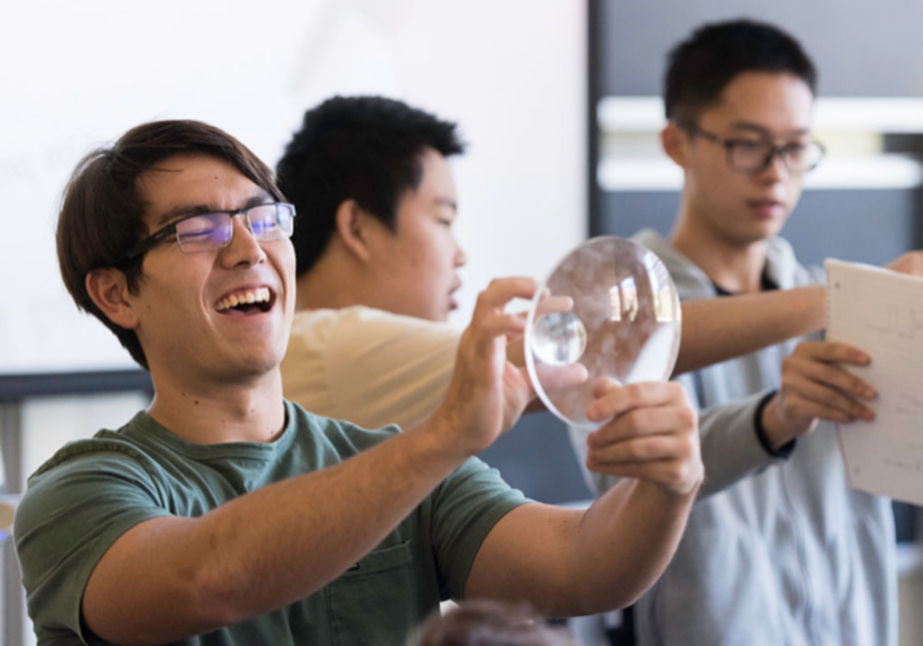
A student admiring a lens in Prof. Brown’s Introductory Optics class

**Figure:**
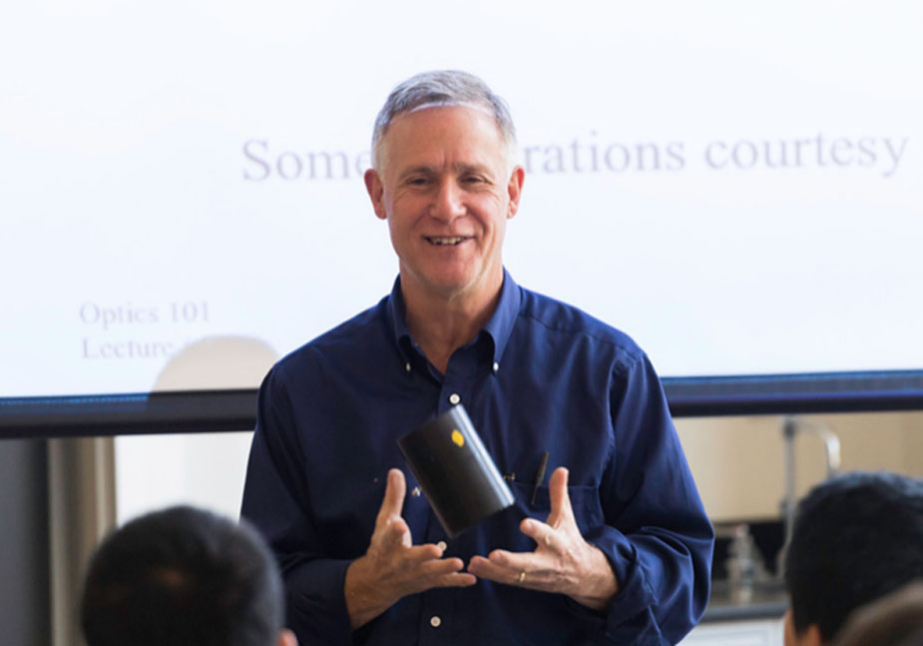
Prof. Brown in the classroom

**Figure:**
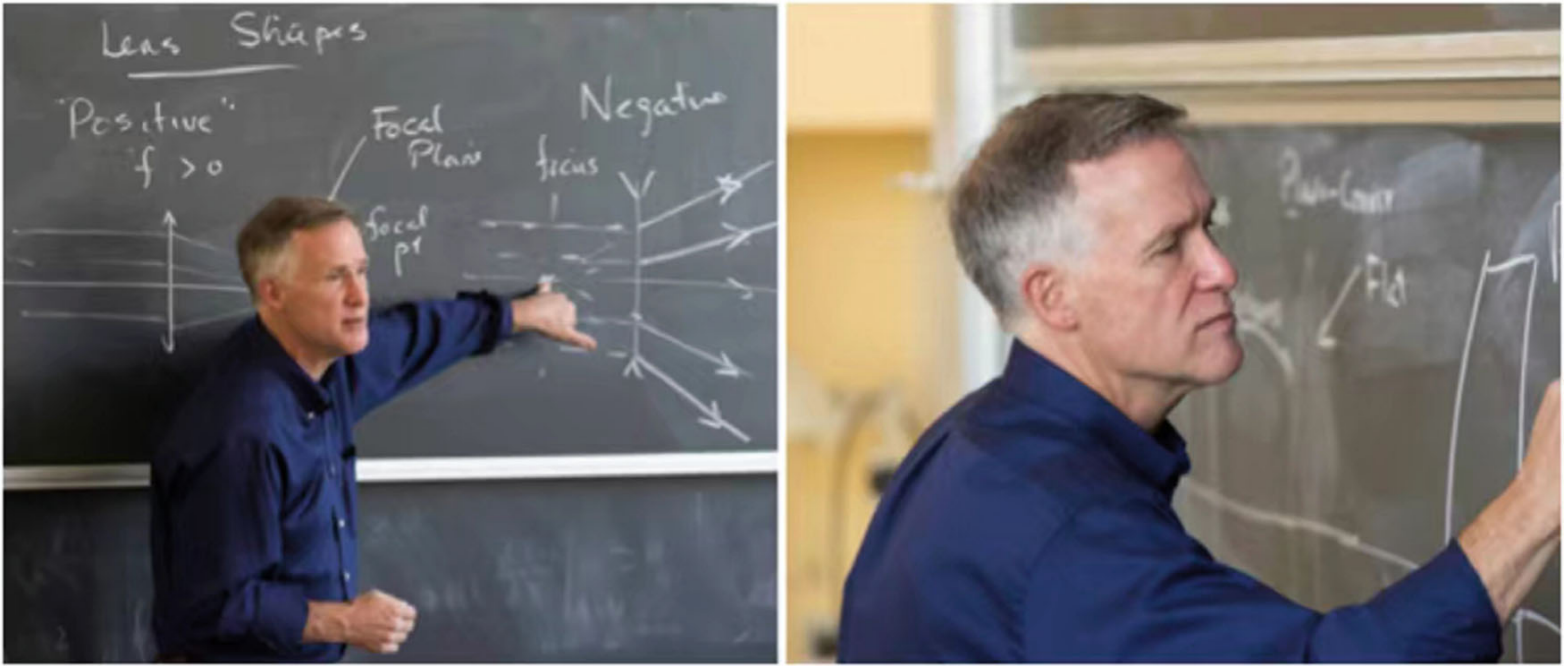
Prof. Brown giving a lecture

**12. Could you share with us about some difficulties or challenges which you find really unforgettable in your career? How did you overcome them?**

Prof. Brown: There are a few scientific efforts I’ve undertaken that I did because they were interesting. But at the time it didn’t seem like many people were interested. For example, I spent my PhD dissertation work looking for ways to make an efficient light emitter from crystalline silicon. This was part of a larger group effort under the supervision of Prof. Dennis Hall, who was convinced that waveguides and devices integrated in and on silicon (similar to microelectronics) was the most sensible and likely future for integrated optics/optoelectronics/photonics. At the time, few people outside of Rochester shared this view and so it was not a hot topic for funding. For the past 20 years, silicon photonics has been very popular, attracting billions of dollars of research funding worldwide, with successful foundries having been established both by government and commercial organizations. Over 30 years later, efficient light emission in crystalline silicon remains a very challenging, largely unsolved problem but the overall future of silicon photonics looks very bright indeed.

**13. Why did you choose optics as your research subject? What kind of career do you think you would have if you haven’t become a scientist?**

Prof. Brown: I was first interested in mathematics as an undergraduate student, but switched to physics based both on the high quality instruction and advising and also based on the interesting applications of some of the mathematics I loved. During my undergraduate years, one of my physics professors spent a sabbatical semester at Bell Labs, studying the emerging world of Fiber Optics. He came back, taught us about gradient index fibers, and I remained fascinated. I was later accepted to a summer research program at GTE Laboratories (Waltham, MA) with a research group working on designing the first live-traffic optical fiber communication systems. I later accepted a full time job with the same group, and realized that I’d like to build a career in optics. After this many years, it is difficult to imagine something other than optics, but I suppose a different path might have taken me to do physics or possibly mathematics.

**14. What is the biggest turning point in your career?**

Prof. Brown: There have been several. I remember, while changing planes in Pittsburgh airport, I sketched out a scheme on a scrap piece of paper that became a method of transforming an ordinary, linearly polarized laser beam into one having radial or azimuthal polarization. We were able to incorporate this into a confocal microscope setup. That was my entry point into the world of polarization and it has been a wonderful experience. Another was my re-entry into silicon photonics by leading the Test, Assembly and Packaging effort within AIM Photonics, a decision that has opened some wonderful doors and connected me with some fantastic people around the world.

**15. In your career, has anyone had a major influence on you? In what way?**

Prof. Brown: My advisor, mentor and director of the Institute, Dennis Hall, perhaps had the biggest personal impact through his example of scholarship, his patience with my development as a faculty member and researcher.

**16. How do you balance your work and family life?**

Prof. Brown: During my early years as a Professor, my children were young and I limited travel to just a few conferences each year. My wife and I made it a priority to have dinner together and communicate with our children’s teachers. We home schooled our children for a few years, and I was able to work with my kids on math and science.

**17. What are your hobbies?**

Prof. Brown: I like very much to canoe and fly fish, and enjoy the outdoors in general, especially in Maine.

**18. What advice and suggestions would you give our young audience on life and career?**

Prof. Brown: Work hard, be patient, and learn as much as you can from the older generation. We advance personally and scientifically from the examples of those who have gone before us.

**Light special correspondent**

*WANG Hui is the Deputy Director of Division of International Cooperation in the Changchun Institute of Optics, Fine Mechanics and Physics (CIOMP), Chinese Academy of Sciences (CAS). She currently works on international communication and cooperation for the CIOMP and was a founding member of the journal Light: Science & Applications, which is a joint publication of Nature Publishing Group and CIOMP. She has published several articles in Acta Editologica, International Talent, Light: Science & Applications, etc., and was invited to contribute an article to SPIE Women in Optics in 2015. She is the initiator of the Rose in Science event and the co-sponsor and moderator of the iCANX Story. She has interviewed Donna Strickland, Nobel Laureate in Physics; Jean-Marie Lehn, Nobel Laureate in Chemistry; Johanna Stachel, the first female president of the German Physical Society; Chennupati Jagadish, president of the Australian Academy of Sciences; Carmen Menoni, president of the IEEE Photonics Society; Lin Li, Academician of the Royal Academy of Engineering; Zhonglin Wang, the first Chinese to receive the Eni Prize, etc.*

